# Narciclasine attenuates sepsis-induced myocardial injury by modulating autophagy

**DOI:** 10.18632/aging.203078

**Published:** 2021-05-25

**Authors:** Rong Tang, Liu Jia, Yunlong Li, Junbo Zheng, Pingping Qi

**Affiliations:** 1 Department of Critical Care Medicine, The Second Affiliated Hospital of Harbin Medical University, Harbin 150086, Heilongjiang Province, China; 2 Departments of Blood Transfusion, The First Affiliated Hospital of Harbin Medical University, Harbin 150086, Heilongjiang Province, China

**Keywords:** acute myocardial injury, narciclasine, autophagy, JNK signaling pathway

## Abstract

Acute myocardial injury (AMI) is often secondary to sepsis, which is a life-threatening disease associated with severe cardiac inflammation. Narciclasine, a plant alkaloid isolated from different members of the *Amaryllidaceae* family, has been extensively characterized as an antitumor and anti-inflammatory compound. In addition, autophagy is critical for sepsis-induced myocardial injury. However, the role and mechanism of autophagy by which narciclasine confers cardioprotection are still unclear. The present study aimed to investigate the underlying mechanism by which narciclasine affects the pathogenesis of sepsis-induced myocardial injury. Narciclasine effectively attenuated LPS-induced myocardial inflammation *in vitro* and *in vivo*. In addition, narciclasine protected cardiac function and suppressed the expression of inflammatory cytokines in LPS-induced heart tissue. Furthermore, narciclasine upregulated LPS-induced autophagic activity, and the autophagy inhibitor 3-MA abrogated narciclasine-mediated protection against LPS-induced AMI. Importantly, narciclasine exerted an inhibitory effect on the JNK signaling pathway, and JNK activity was tightly associated with narciclasine-induced autophagy and the consequent protective effects during AMI. Taken together, our findings indicate that narciclasine protects against LPS-induced AMI by inducing JNK-dependent autophagic flux; hence, narciclasine may be an effective and novel agent for the clinical treatment of sepsis-induced myocardial injury.

## INTRODUCTION

Sepsis is a systemic inflammatory response syndrome and the main cause of death in critically ill patients [1, 2]. Cardiac dysfunction with left ventricular (LV) dilation and reduced LV ejection fraction (LVEF) accompanied by sepsis is the main cause of death in intensive care units. The infectious organisms and dead or damaged cells released pathogen-related molecular patterns and damage-related molecular patterns (DAMPs) activate inflammatory responses. Overproduction of inflammatory cytokines including tumor necrosis factor (TNF)-α, interleukin (IL)-1α, IL-1β, and IL-6 also leads to cardiac dysfunction in sepsis [3–5]. Treatments for septic cardiomyopathy include optimizing volume loading and blood pressure, improving cardiac contractility, and alleviating inflammation. Inhibiting inflammation is considered a potential therapeutic strategy for sepsis-induced cardiomyopathy [6–8].

Accumulating evidence demonstrates that autophagy is involved in the regulation of inflammatory responses, in different cell types in the heart and thus plays crucial roles in sepsis-induced cardiomyopathy [9–11]. So far, the effects of autophagy in the pathogenesis of sepsis remain unclear. The effects of autophagy on inflammatory cardiac diseases could be protective or adverse depending on the different conditions [12–15]. On the one hand, autophagy controls cardiac inflammatory responses by limiting the inflammasome activators and/or components [16–18] and by decreasing DAMPs from mitochondria [19]. On the other hand, overactive autophagy in the context of myocardial infarction or ischemia/reperfusion stress may exacerbate adverse results due to the degradation of cellular components, leading to a positive inflammatory response [20].

Natural products from plants and their derivatives are valuable sources of therapeutic agents [21, 22]. Plants in the *Amaryllidaceae* family are known for their pharmacologically active alkaloids. Traditionally, corm extracts from chicory bulbs have been used to treat inflammation-related diseases [23]. Narciclasine is an isocyanide alkaloid that is present in the bulbs of Coneflower extract and has been found to have estrogen-killing properties [24]. Narcissus has been shown to improve the prognosis of neonatal rats with sepsis by inhibiting calprotectin and reducing inflammation [25, 26]. Narciclasine has also been shown to improve outcomes in septic neonatal rats by alleviating inflammatory responses [27].

The activation of multiple stress signaling cascade like mitogen-activated protein kinase (MAPK) is believed to play a key role in sepsis-induced cardiomyopathy [28, 29]. C-jun N-terminal kinase (JNK) is a member of the MAPK family that is important in numerous human diseases, including sepsis-induced cardiomyopathy [30, 31]. Autophagy plays an important role in inflammation-induced AMI. However, the role of autophagy and the mechanism of narciclasine-afforded cardioprotection remain unclear. In this study, we aimed to study whether narciclasine could improve LPS-induced AMI by regulating autophagy and whether the regulation of autophagy in AMI is mediated by the JNK signaling pathway.

## RESULTS

### Narciclasine attenuates LPS-induced cardiomyocyte inflammation

**Figure 1:**
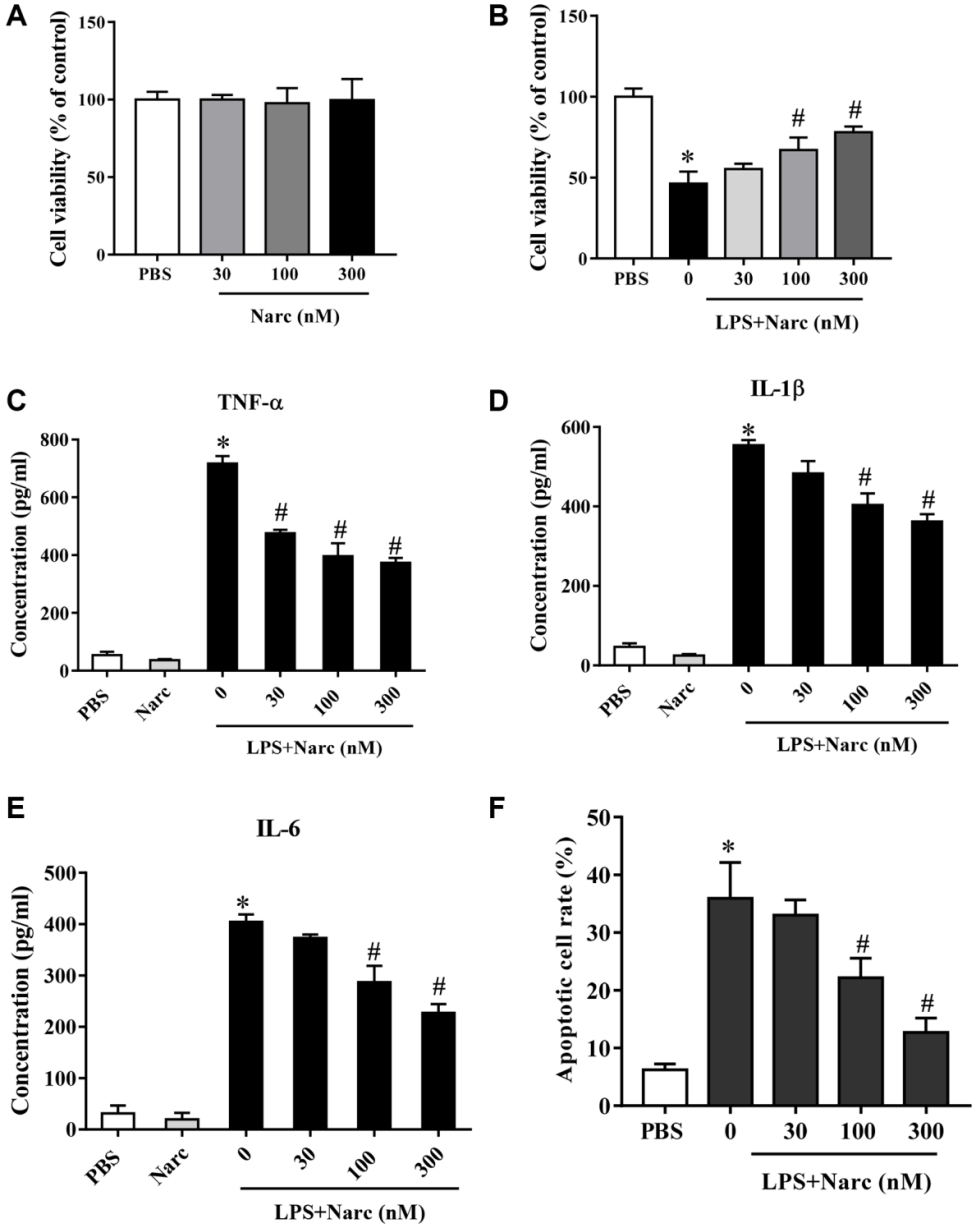
**Narciclasine attenuates LPS-induced cardiomyocyte inflammation.** (**A**) The viability of cardiomyocytes treated with different concentrations of narciclasine (0, 30, 100, and 300 nM) was measured by CCK8 assays. (**B**) The viability of cardiomyocytes stimulated with LPS and treated with different concentrations of narciclasine (0, 30, 100, and 300 nM) was measured by CCK8 assays. (**C**–**E**) The concentration of inflammatory cytokines (TNF-α, IL-1β, and IL-6) in cardiomyocytes stimulated with LPS and treated with different concentrations of narciclasine (0, 30, 100, and 300 nM) was measured by ELISA kits. (**F**) Quantification of LPS-induced neonatal rat cardiomyocyte apoptosis was analyzed by FACS. *n* = 3 per group. Data represent the mean ± SEM. ^*^P < 0.05 vs. the PBS group. ^#^*P* < 0.05 vs. the LPS group.

We stimulated isolated neonatal rat cardiomyocytes (NRCMs) with LPS and treated these NRCMs with narciclasine (0, 30, 100, and 300 nM). Cell viability of NRCMs was not affected by narciclasine (Figure 1A), but the LPS-induced decrease in cell viability was attenuated by narciclasine treatment in a dose-dependent manner (Figure 1B). In addition, narciclasine suppressed LPS-induced inflammatory cytokine (TNF-α, IL-1β, and IL-6) release from NRCMs in a dose-dependent manner (Figure 1C–1E). Narciclasine also inhibited LPS-induced apoptosis in NRCMs in a dose-dependent manner (Figure 1F). These data indicated that narciclasine inhibited the LPS-induced inflammation in cardiomyocytes.

### Narciclasine attenuates LPS-induced myocardial dysfunction

**Figure 2:**
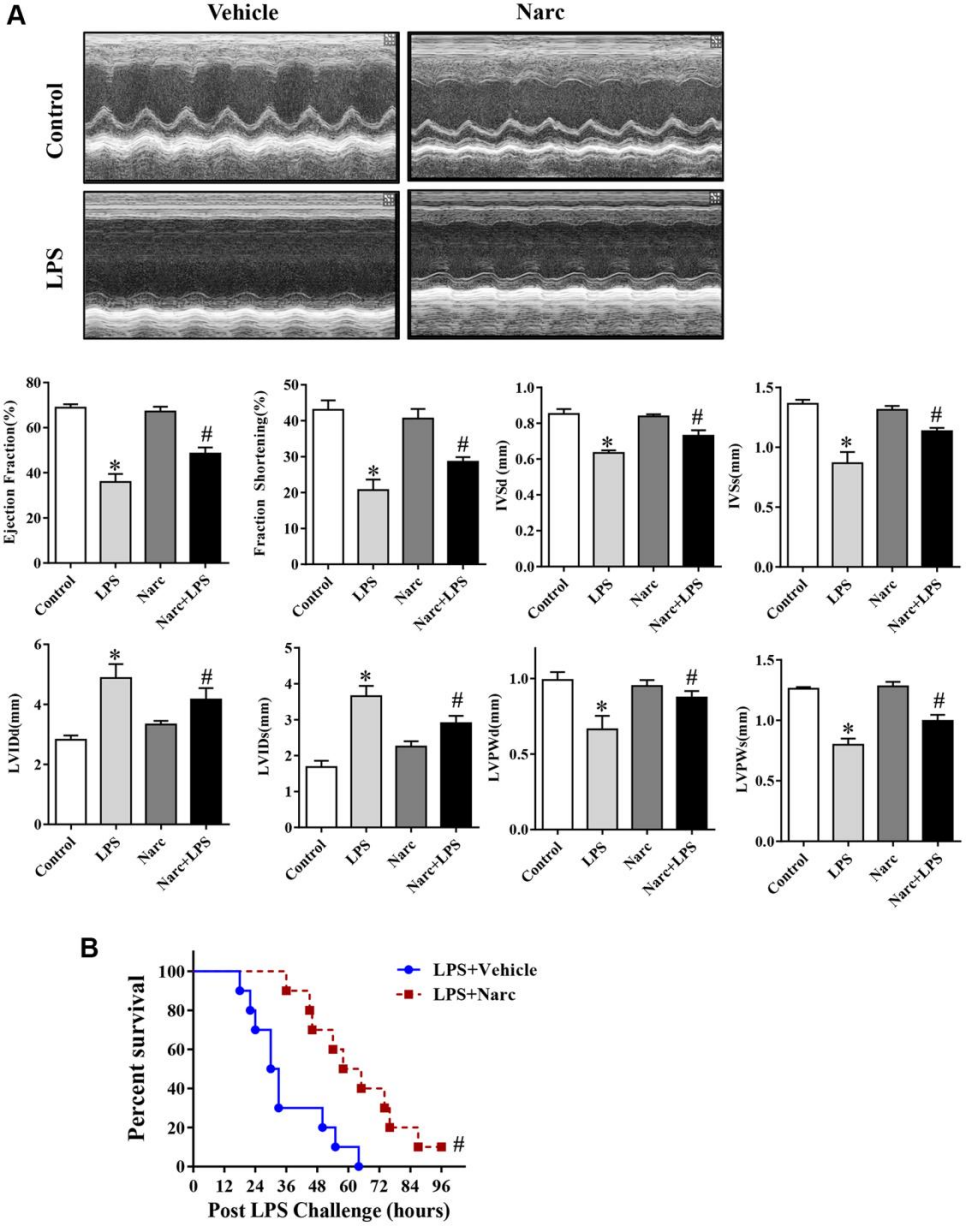
**Narciclasine attenuates LPS-induced myocardial dysfunction.** (**A**) Echocardiographic analysis of LVEF, LVFS, IVSd, IVSs, LVIDs, LVIDd, LVPWd, and LVPWs after LPS challenge for 12 h. (**B**) The survival rates of LPS-injected mice throughout the 96-h study period. LVEF, left ventricular ejection fraction; LVFS, left ventricular fractional shortening; IVSd and IVSs, interventricular septal end diastole and end systole, respectively; LVIDd and LVIDs, left ventricular internal diameter end diastole and end systole, respectively; LVPWd and LVPWs, left ventricular posterior wall end diastole and end systole, respectively. *n* = 8 per group. The data are shown as the means ± SEM. ^*^P < 0.05 vs. the PBS group. ^#^*P* < 0.05 vs. the LPS group.

To investigate the role of narciclasine in LPS-induced AMI, mice were administered narciclasine before LPS injection. Echocardiography was performed to evaluate cardiac functions. There was no significant difference between the control group and narciclasine groups in the absence of LPS stimulation. After 12 h of LPS stimulation, the mice showed significant cardiac dysfunction, as evidenced by increased LVIDs and LVIDd, decreased ejection fraction, and fractional shortening compared with those of the control group. Interestingly, narciclasine treatment (LPS + Narc) partly improved these parameters (Figure 2A). In addition, treatment with narciclasine elevated the survival rates of LPS-induced mice (Figure 2B).

### Narciclasine attenuates the LPS-induced inflammatory response *in vivo*

**Figure 3:**
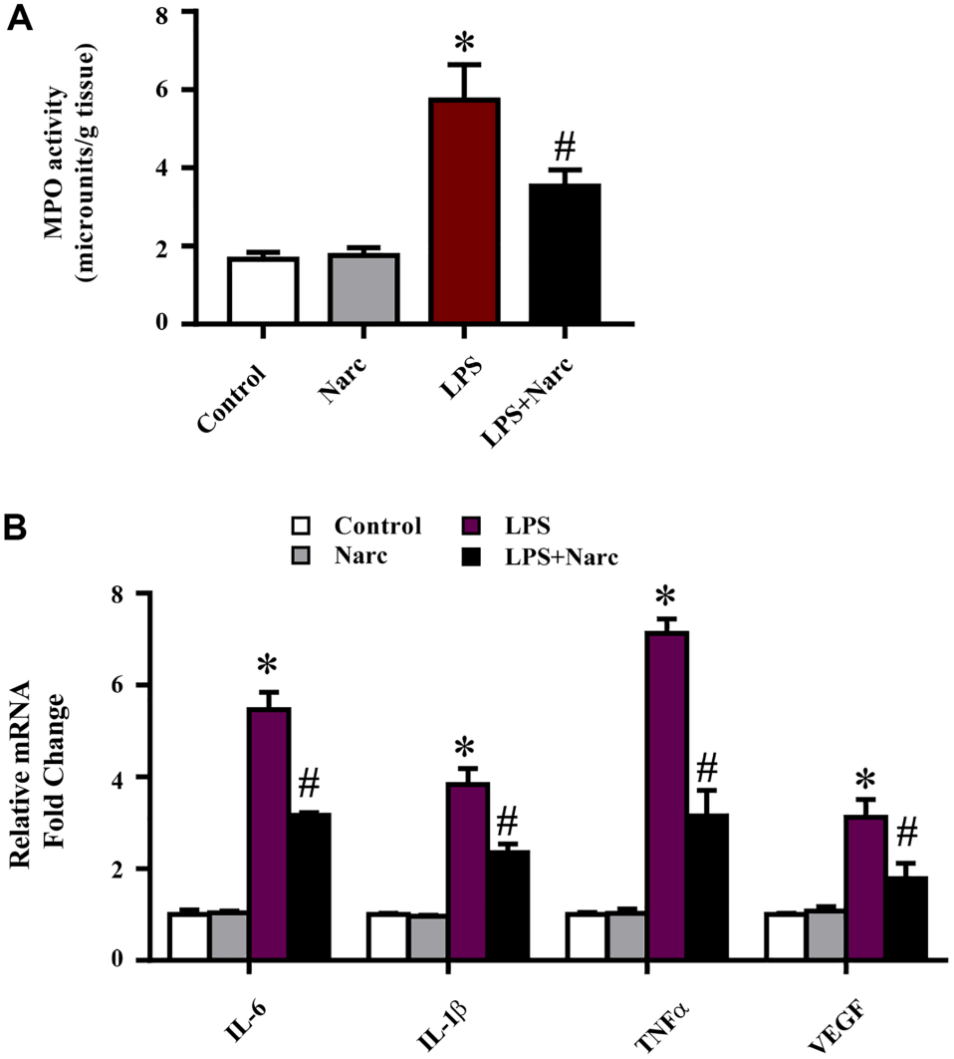
**Narciclasine attenuates the LPS-induced inflammatory response *in vivo*.** (**A**) Myeloperoxidase (MPO) activity was measured by an MPO assay kit. (**B**) The mRNA expression levels of IL-6, IL-1β, TNF-α, and VEGF in heart tissues were measured by q-PCR. *n* = 3 per group. The data are shown as the means ± SEM. ^*^P < 0.05 vs. the PBS group. ^#^*P* < 0.05 vs. the LPS group.

To evaluate the cardioprotective effects of narciclasine on inflammation in LPS-induced AMI, the levels of inflammatory indicators were measured. An indicator of neutrophil infiltration, MPO, was increased in the LPS group, and narciclasine effectively abrogated this increase (Figure 3A). Additionally, the mRNA expression levels of IL-6, IL-1β, TNF-α, and VEGF in heart tissues were significantly increased in LPS-induced AMI but were notably attenuated by narciclasine treatment (Figure 3B). These results demonstrated that narciclasine could attenuate heart inflammation in LPS-induced AMI.

### Autophagy is upregulated during narciclasine-mediated attenuation of LPS-induced AMI

**Figure 4:**
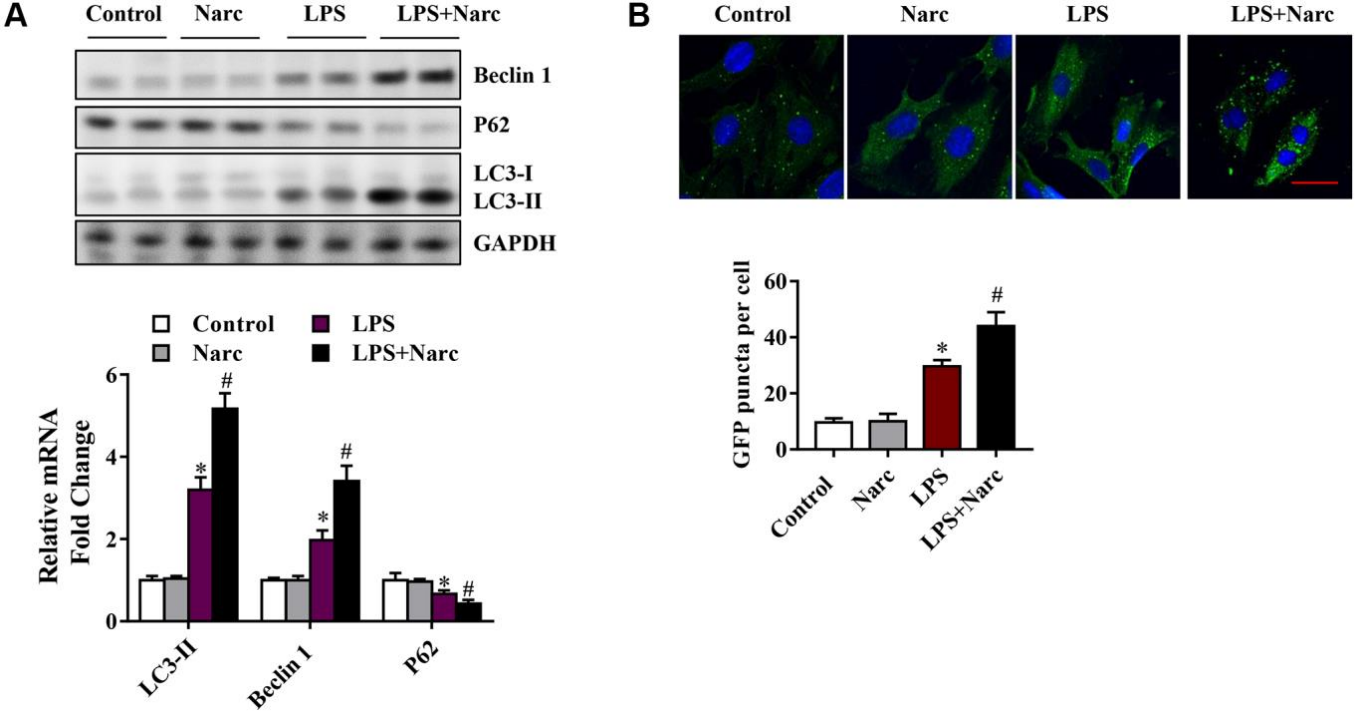
**Narciclasine upregulates autophagy in a mouse model of LPS-induced myocardial injury.** (**A**) Western blot analysis of autophagy markers, including Beclin-1, p62 and microtubule-associated light chain (LC)-3, in heart tissue. (**B**) The autophagy level was measured by live-cell imaging using Ad-GFP-LC3. Scale bar, 20 μm. *n* = 3 per group. The data are shown as the means ± SEM. ^*^P < 0.05 vs. the PBS group. ^#^*P* < 0.05 vs. the LPS group.

To determine whether narciclasine could activate autophagy in LPS-induced AMI, we examined several autophagy indicators, such as microtubule-associated light chain (LC)-3, p62, and Beclin-1. Beclin-1 and LC3-II were increased while the p62 expression level was decreased in the LPS group compared with the sham group (Figure 4A). However, narciclasine administration further upregulated Beclin-1 and LC3-II expression and further downregulated p62 expression in response to LPS-induced AMI (Figure 4A). Furthermore, autophagy was measured by live-cell imaging using a GFP-LC3 adenovirus (Figure 4B). We found that narciclasine treatment upregulated the autophagy level, as indicated by increased GFP+ puncta. These data indicated that narciclasine could improve autophagy in the heart during LPS-induced AMI.

### Autophagy inhibition abrogates narciclasine-mediated protection against LPS-induced myocardial injury

**Figure 5:**
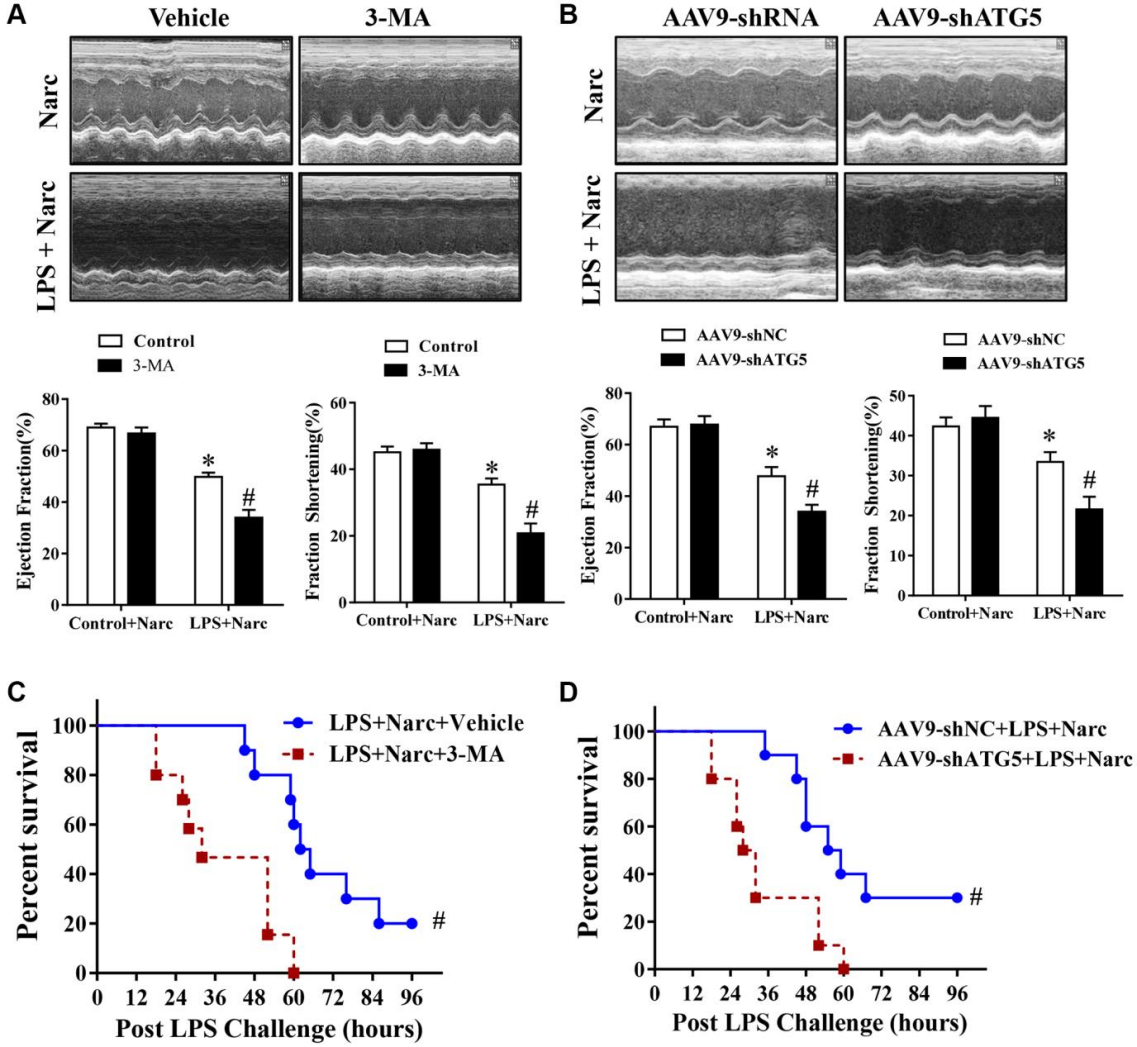
**The autophagy inhibitor 3-MA and knockdown of ATG5 abrogates narciclasine-mediated protection against LPS-induced myocardial injury.** (**A**–**B**) Echocardiographic analysis of the LVEF and LVFS in mouse hearts after LPS challenge for 12 h. (**C**–**D**) The survival rates of LPS-injected mice throughout the 96-h study period. *n* = 8 per group. The data are shown as the means ± SEM. *P < 0.05 vs. the Narc group. ^#^*P* < 0.05 vs. the Narc+LPS group.

To study the effects of narciclasine-induced autophagy in AMI model, C57BL/6 male mice were pretreated with autophagy inhibitor 3-methyladenine (3-MA) before LPS administration. Echocardiography was performed to evaluate heart functions. Based on the ejection fraction and fractional shortening parameters, 3-MA did not influence heart function in normal hearts but abrogated the protective effect of narciclasine on LPS-induced myocardial injury (Figure 5A). In addition, 3-MA increased the mortality rate in narciclasine-pretreated LPS-induced mice (Figure 5C). Similar results were found in mice in which endogenous ATG5 was knocked down, which is a genetic strategy that interferes with autophagy (Figure 5B and 5D). Taken together, these results indicated that narciclasine has cardioprotective effects in LPS-induced AMI via autophagy.

### The JNK signaling pathway regulates narciclasine-mediated protection against sepsis-induced myocardial injury

**Figure 6:**
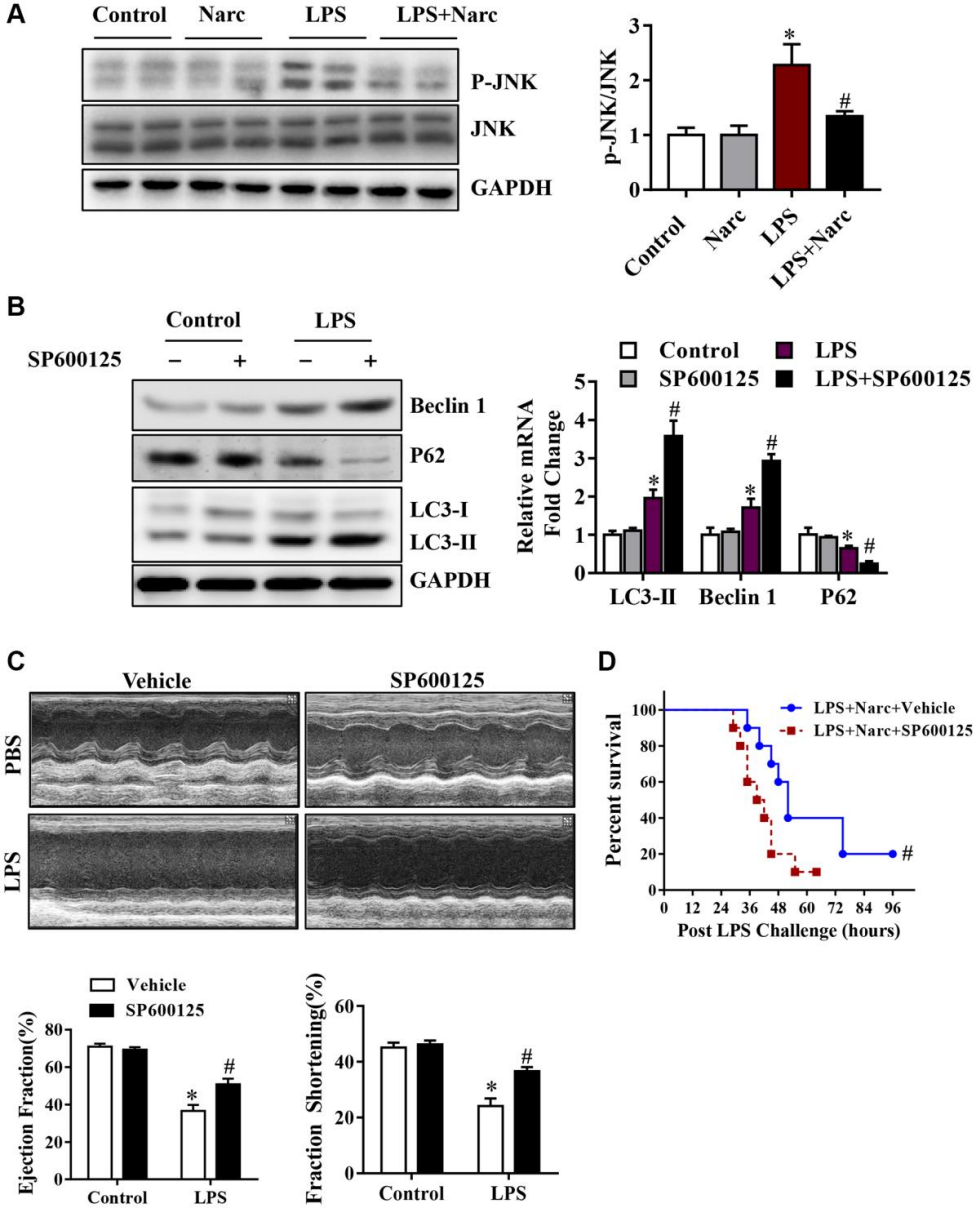
**The JNK signaling pathway is responsible for narciclasine-mediated protection against sepsis-induced myocardial injury.** (**A**) The expression levels of total JNK and phosphorylated JNK (p-JNK) were measured by western blotting. Mice were treated with SP600125, an inhibitor of JNK activity, prior to LPS stimulation to further investigate the role of JNK in autophagy. (**B**) The levels of autophagy-associated proteins, p-JNK and JNK were assessed by western blotting. (**C**) The LVEF and LVFS of mouse hearts after LPS challenge for 96 h were measured by echocardiography. (**D**) The survival rates of LPS-injected mice throughout the 96-h study period. *n* = 3–8 per group. The data are shown as the means ± SEM. ^*^P < 0.05 vs. the PBS group. ^#^*P* < 0.05 vs. the LPS group.

To determine the role of JNK signaling pathway in narciclasine-induced autophagy, we measured the phosphorylation of JNK. Interestingly, LPS treatment significantly increased the phosphorylation of JNK while narciclasine treatment reduced the phosphorylation by LPS (Figure 6A). SP600125, an inhibitor of JNK activity, was treated on mice prior to LPS stimulation to further study the contribution of JNK signaling pathway in autophagy. We found that SP600125 treatment suppressed the phosphorylation of JNK and increased the autophagy-associated proteins expression (Figure 6B). Additionally, SP600125 pretreatment ameliorated heart function and improved the survival rates of LPS-induced AMI mice (Figure 6C–6D). The above findings indicated that narciclasine-induced autophagy was mediated by inhibiting the phosphorylation of JNK.

### Narciclasine-induced autophagy is mediated by JNK signaling pathway in cardiomyocytes

**Figure 7:**
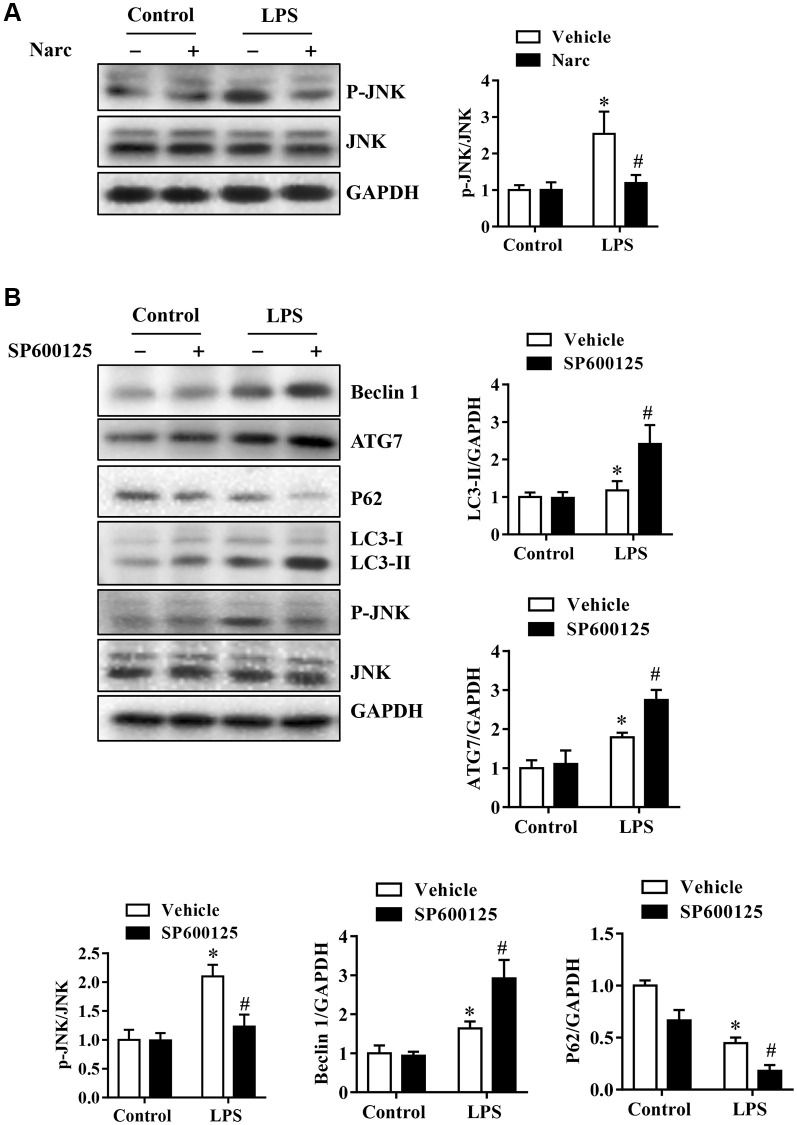
**Narciclasine-induced autophagy is associated with JNK activity in cardiomyocytes.** Neonatal rat cardiomyocytes were pretreated with narciclasine (300 nmol/L, 30 min) or SP600125 (SP, 20 μmol/L, 30 min) before LPS challenge. (**A**) JNK activity in the presence or absence of narciclasine was measured by western blotting. (**B**) The levels of autophagy-associated proteins, p-JNK, and JNK were examined by western blotting. *n* = 3 per group. The data are shown as the means ± SEM. ^*^P < 0.05 vs. the PBS group. ^#^*P* < 0.05 vs. the LPS group.

To investigate the mechanism of narciclasine in AMI, we stimulated NRCMs with 1000 ng/ml LPS to simulate the AMI model *in vitro*. SP600125 is also used on NRCMs to inhibit JNK activity, thereby explain the regulatory role of JNK in autophagy. Narciclasine decreased JNK phosphorylation levels in response to LPS stress in NRCMs, which was similar to the effect in the *in vivo* LPS-induced AMI model (Figure 7A–7B). Besides, the phosphorylation of JNK was inhibited by SP600125, while LC3B-II/Iand Beclin-1 levels were increased and p62 levels were decreased in NRCMs (Figure 7A–7B). We also confirmed this result by live-cell imaging using a GFP-LC3 adenovirus and found that SP600125 increased the autophagy level compared to that in the LPS group. These results demonstrated that inhibition of JNK could enhance autophagic activity in cardiomyocytes.

## DISCUSSION

Narciclasine is an isocyanide alkaloid that is present in the bulbs of coneflower extract and has been shown to have estrogen-killing properties. Narciclasine has also been shown to improve the prognosis of neonatal rats with sepsis by reducing inflammation [25–27]. In the present study, we evaluated the role and the underlying mechanism of narciclasine on the pathogenesis of AMI. We demonstrated that narciclasine attenuated LPS-induced cardiomyocyte inflammation, protected heart function and suppressed inflammation in LPS-induced AMI. Mechanistically, narciclasine protected against LPS-induced AMI by inducing JNK-dependent autophagy.

The heart is an important organ that is frequently affected by sepsis [32–34]. Sepsis-induced systemic inflammatory syndrome is a result of the combined actions of PAMPs (pathogen-associated molecular patterns) and DAMPs, which initiate the overwhelming production of inflammatory cytokines [35, 36]. LPS triggers inflammatory cytokine secretion, resulting in the release of DAMPs from activated, injured, or necrotic cells [37, 38]. The LPS-induced AMI model was treated with or without narciclasine, and we measured the expression of inflammatory cytokines and found that narciclasine inhibited neutrophil infiltration and decreased the expression of IL-6, IL-1β, and TNF-α. Our results indicated that narciclasine attenuates cardiac inflammation in LPS-induced AMI.

Multiple researches indicate that sepsis initiates autophagy in multiple organs, including the heart. Autophagy is critical for cell survival and death, homeostasis, and development [39, 40]. Evidence from CLP and LPS-induced sepsis models suggests that inducing autophagy pharmacologically protects the heart, providing evidence that autophagy is a cellular adaptive response [9, 41]. In line with these observations, we showed that narciclasine can increase Beclin-1 and LC3B-II level, decrease p62 level, and promote autophagic activity in LPS-induced AMI mice. In order to determine the protective role of narciclasine-induced autophagy, 3-MA was used to inhibit narciclasine-induced autophagy. We found that 3-MA abolished the beneficial effect of narciclasine on LPS-induced AMI in mice by blocking autophagy. These data suggested that narciclasine confers its cardioprotective effects via induction of autophagy.

JNK signaling pathway is important for the regulation of autophagy [42]. It was reported that JNK phosphorylation was increased in sepsis-induced acute lung injury [43, 44]. Our study also showed that in the LPS-induced AMI mouse model, p-JNK levels in the heart increased after LPS stimulation, while narcissus reduced pJNK levels and upregulated autophagy. More importantly, the inhibitory effect of SP600125 on JNK also triggered autophagy and reduced heart damage. Similar findings were observed *in vitro*. The inhibitory effect of SP600125 on JNK further enhanced the effect of autophagy in NRCMs on LPS attack. Although these results indicate that narciclasine-induced autophagy on AMI is mediated by the inhibition of JNK signaling pathway, the mechanism by which narciclasine inhibits JNK still needs further study.

In summary, we demonstrated that narciclasine could alleviate LPS-induced AMI by increasing autophagy, and uncovered the mechanism by which autophagy protects against AMI by the inhibition of JNK signaling pathway. These data suggest that narciclasine acts as a promising agent to protect the heart against sepsis injury, and autophagy may also be a therapeutic target for AMI treatment.

## MATERIALS AND METHODS

### Animals

Adult male C57BL/6 mice were used in the experiments. All procedures involving animals were performed in accordance with the Guidelines of the National Institutes of Health for Animal Care and Use and were approved by the Institutional Animal Care and Use Committee and Ethics Committee of Harbin Medical University. The saline injected mice or the LPS-treated mice (a single 6 mg/kg injection of LPS) were pretreated with or without narciclasine (0.1 mg/kg body weight) administered by gavage for 7 days. Control mice were given equal volumes of saline by gavage. The mice were killed, and the hearts were collected after 6 hours.

### Echocardiography

Transthoracic echocardiography (VEVO 2100, Visual Sonics) with a 25-MHz imaging transducer was performed on anesthetized animals as previously described [18]. Left ventricular (LV) ejection fraction (LVEF), LV fractional shortening (LVFS), interventricular septal end diastole and end systole (IVSd, IVSs), LV systolic dimension (LVDs), LV posterior wall diastolic dimensions (LVPWd) and LV posterior wall systolic dimensions (LVPWs) were measured.

### Isolation and culture of NRCMs

The isolation and culture of NRCMs were performed as described previously [7]. Briefly, cardiomyocytes were collected from the freshly dissected ventricles of 1–3-day-old Sprague Dawley rats were plated on gelatin-coated dishes in Dulbecco’s modified Eagle’s medium, 20% M199, 15% FBS and 1% penicillin/streptomycin (P/S). The cells were transferred to low-serum maintenance media containing DMEM, 18.5% M199, 5% horse serum, 1% P/S after 24 hours and maintained at 37°C and 5% CO_2_ in a humidified environment.

### Real-time polymerase chain reaction

The real-time PCR was performed as described previously [20]. The following primer sequences were used: TNF-α, forward: CATCTTCTCAAAATTCGAGTGACAA, reverse: TGGGAGTAGACAAGGTACAACCC; IL-1β, forward: GTGGCTGTGGAGAAGCTGTG, reverse: GAAGGTCCACGGGAAAGACAC; IL-6, forward: GAGGATACCACTCCCAACAGACC, reverse: AAGTGCATCATCGTTGTTCATACA; and GAPDH, forward: TGACCTCAACTACATGGTCTACA, reverse: CTTCCCATTCTCGGCCTTG.

### Western blotting

The heart tissues and neonatal rat cardiomyocytes proteins were extracted with RIPA lysis buffer (containing protease and phosphatase inhibitors, Thermo Scientific), after which the lysate was centrifuged at 12,000 rpm, 30 min at 4°C. The protein supernatant was then collected and analyzed by standard immunoblotting. Protein samples were prepared for PAGE after the concentrations were measured by a BCA assay (Thermo Scientific). The proteins were then transferred to a PVDF membrane (0.45 μm, BioRad) and probed with the indicated antibodies. The primary antibodies used were Beclin 1 (3738S, Cell Signaling Technology), P62 (23214, Cell Signaling Technology), LC3 (ab192890, Abcam), JNK (ab199380, Abcam), p-JNK (ab215208, Abcam) and GAPDH (2118, Cell Signaling Technology).

### Enzyme-linked immunosorbent assay (ELISA) and myeloperoxidase (MPO) activity measurement

TNF-α, IL-1β, and IL-6 levels in cardiomyocyte lysates were quantitatively measured by commercial ELISA kits according to the manufacturer's instructions (ABclonal, Wuhan, China; Dakewe, Shenzhen, China). MPO activity in heart lysates was measured according to the manufacturer's procedure (Nanjing Jiancheng Corp., Nanjing, China).

### Quantification of apoptosis by fluorescence-activated cell sorting (FACS)

The high affinity of Annexin-V (AV) for phosphatidylserine (PI) (Beyotime, China), which is exposed on the surface of apoptotic cells, was used to examine apoptosis as described previously [27]. The data are reported as the percentages of early apoptotic cells (FITC^+^/PI^-^) and late apoptotic cells (FITC^+^/PI^+^).

### AAV9 vector generation and transfection

We prepared AAV9-shNC and AAV9-shATG5 vectors as described [45]. We injected 4- to 5-week-old male C57 mice with 1 × 10^12^ vg of AAV9-shNC and AAV9-shATG5 intravenously via the tail vein as described previously [46].

### Statistical analysis

Results are expressed as the mean ± standard error of the mean (SEM). GraphPad Prism 8.0 (GraphPad Software Inc., CA, USA) is used to determine the statistical significance among multiple groups by one-way ANOVA with the Bonferroni post hoc test. A 2-tailed *p*-value of less than 0.05 was considered statistically significant.
